# Visual attention for a desktop virtual environment with ambient scent

**DOI:** 10.3389/fpsyg.2013.00883

**Published:** 2013-11-26

**Authors:** Alexander Toet, Martin G. van Schaik

**Affiliations:** ^1^TNOSoesterberg, Netherlands; ^2^Department of Information and Computing Sciences, University UtrechtUtrecht, Netherlands

**Keywords:** attention, ambient odor, semantic congruency, affective congruency, virtual environment

## Abstract

In the current study participants explored a desktop virtual environment (VE) representing a suburban neighborhood with signs of public disorder (neglect, vandalism, and crime), while being exposed to either room air (control group), or subliminal levels of tar (unpleasant; typically associated with burned or waste material) or freshly cut grass (pleasant; typically associated with natural or fresh material) ambient odor. They reported all signs of disorder they noticed during their walk together with their associated emotional response. Based on recent evidence that odors reflexively direct visual attention to (either semantically or affectively) congruent visual objects, we hypothesized that participants would notice more signs of disorder in the presence of ambient tar odor (since this odor may bias attention to unpleasant and negative features), and less signs of disorder in the presence of ambient grass odor (since this odor may bias visual attention toward the vegetation in the environment and away from the signs of disorder). Contrary to our expectations the results provide no indication that the presence of an ambient odor affected the participants’ visual attention for signs of disorder or their emotional response. However, the paradigm used in present study does not allow us to draw any conclusions in this respect. We conclude that a closer affective, semantic, or spatiotemporal link between the contents of a desktop VE and ambient scents may be required to effectively establish diagnostic associations that guide a user’s attention. In the absence of these direct links, ambient scent may be more diagnostic for the physical environment of the observer as a whole than for the particular items in that environment (or, in this case, items represented in the VE).

## INTRODUCTION

### BACKGROUND

Desktop virtual environments (VEs) are increasingly deployed to study future design plans and the possible effects of environmental qualities and interventions on human behavior and feelings of safety in built environments with signs of public disorder (Cozens et al., 2003; Park et al., 2008, 2010; Toet and van Schaik, 2012). Desktop VEs offer cost-effective, safe, controlled, and flexible environments that allow to investigate human response to a wide range of environmental factors without the constraints, distractions, and dangers of the real world (e.g., Nasar and Cubukcu, 2011). They are relatively cheap, widely available, and easy to use, while most users are familiar with these displays and their interaction devices. Desktop VEs are also preferred for communication of design and intervention plans because they can be made accessible to a large numbers of users in internet applications (Dang et al., 2012). For these applications it is essential that users perceive the desktop VE in a similar way as they would perceive its real world counterpart. Previous studies have shown that environmental characteristics like lighting, sound, and dynamic elements similarly affect the perception of desktop VEs and real environments (Bishop and Rohrmann, 2003; Houtkamp et al., 2008). Ambient scent is another important environmental characteristic that is currently lacking in most VEs. Ambient scent is known to significantly affect our perception of real environments (Wrzesniewski et al., 1999), and people have strong expectations about the way an environment should smell (Henshaw and Bruce, 2012). It has also been shown that ambient odor can increase the sense of presence in immersive VEs (Dinh et al., 1999; Washburn et al., 2003; Tortell et al., 2007). Thus, ambient odors may be an effective tool to tune the user perception of less immersive desktop VEs (e.g., by evoking implicit associations).

Despite the importance of scent in our everyday life olfaction is rarely applied in the scope of VEs (Baus and Bouchard, 2010). Recent technological developments enable the effective and localized dispersion and control of scents (Yanigada et al., 2003, 2004, 2005; Yu et al., 2003; Oshima et al., 2007; for reviews see Richard et al., 2006; Riener and Harders, 2012), thereby providing VE researchers and developers with the ability to utilize scent to create compelling VEs (Tomono et al., 2011). Enhancing VEs with olfactory stimuli may enhance user experience by heightening the sense of reality (Chalmers et al., 2009; Ghinea and Ademoye, 2011). It has indeed been shown that the addition of olfactory cues to an immersive VE can increase the user’s sense of presence, memory and perceived realism of the simulated environment (Dinh et al., 1999; Washburn et al., 2003; Tortell et al., 2007). However, it is still unknown if ambient scents can influence the attention for details in a desktop VE (Ghinea and Ademoye, 2011).

In a previous study we found that signs of disorder influence the affective appraisal of a desktop VE to a large degree in a similar way as the appraisal of its real world counterpart (Toet and van Schaik, 2012). However, it appeared that participants focused more on signs of disorder in a desktop VE than in a similar real world environment. This finding, which may seriously degrade the ecological validity of VEs for the aforementioned applications, was partly reduced by the addition of a realistic soundscape to the VE simulation. We argued that in the real world the saliency of signs of public disorder is typically modulated by various environmental factors which are typically lacking in a desktop VE, such as ambient sounds, tactile or olfactory cues. For instance, their saliency may be ameliorated by the sound of birds, a soft warm breeze, sun, and pleasant ambient smells of fresh air and vegetation, or enhanced by loud noise, strong cold wind, or unpleasant (e.g., garbage and urine) smells. In this study we investigated if ambient odors can influence the visual attention for these details in a desktop VE.

### VISUAL-OLFACTORY INTERACTIONS

Interactions between olfaction and vision appear to be widespread. Neuroimaging studies have shown that interaction between olfaction and vision occurs at multiple levels of information processing (Gottfried and Dolan, 2003; Österbauer et al., 2005; Walla, 2008; Seubert et al., 2013). Also, it was found that stimulation of the human visual cortex enhances odor discrimination (Jadauji et al., 2012). Linking the perceptions of odors and colors appears to occur mainly in the amygdala and the orbitofrontal cortex (OFC; Gottfried and Dolan, 2003; Österbauer et al., 2005).

The amygdala is a central perceptual node where information from olfactory, visual, auditory, and tactile modalities converges (Zald, 2003). It is an integral component of a distributed affective circuit in the mammalian brain that mediates both positive and negative affect and the processing of reward-predicting cues (Murray, 2007). Recent evidence suggests that the amygdala also plays a central causal role in the modulation of visual attention (Vuilleumier, 2005; Williams et al., 2005; Duncan and Feldman Barrett, 2007; for a recent overview see Pourtois et al., 2013). The amygdala enhances the visual saliency of affective targets (Duncan and Feldman Barrett, 2007). This implies that the activation state of the amygdala determines whether affective features or objects are prioritized. Since the amygdala responds to both positive and negative valenced odors (but not to neutral odors: Winston et al., 2005), olfactory induced amygdala activity may boost visual attention for affectively congruent (potentially threatening or rewarding) targets (Vuilleumier, 2005; Mohanty et al., 2009; Jacobs et al., 2012).

There is ample evidence for the visual modulation of olfactory perception. A neutral suprathrehold odor is rated significantly more pleasant after viewing positive pictures and significantly less pleasant and more intense after seeing unpleasant pictures (Pollatos et al., 2007). A visual feature that has a particular strong influence on odor perception is color (Zellner, 2013). Color enhances the perceived intensity of odors (independent of color appropriateness: Zellner and Kautz, 1990). Color also modulates the hedonic value of odors: both neural response in brain area encoding the hedonic value of smells (Österbauer et al., 2005) and the subjectively judged pleasantness of color-odor combinations (Zellner et al., 1991) increase with perceived color-odor appropriateness. Odors are detected faster and more accurately in the presence of semantically congruent colors (Zellner et al., 1991) or pictures (Gottfried and Dolan, 2003; Demattè et al., 2009), while incongruent colors and shape cues reduce odor discrimination accuracy (Demattè et al., 2009). Color-smell associations can be so compelling that color can even completely change the quality of the perceived odor (a white wine is perceived as having the odor of a red wine when artificially colored red: Morrot et al., 2001). Visual-olfactory interactions appear to be automatic: color and shape cues affect the accuracy of odor discrimination, even when the information is task irrelevant and when participants are explicitly instructed to ignore these cues (Demattè et al., 2009). Specific odor components of complex odor mixtures that are congruent with a presented color are perceived as more prominent, suggesting that color directs olfactory attention to color-associated components (Arao et al., 2012). Functional magnetic resonance imaging studies have shown neurophysiological correlates of olfactory response modulation by color cues: activity in caudal regions of the OFC and in the insular cortex increase progressively with perceived odor-color congruency (Österbauer et al., 2005).

In contrast to the large amount of evidence for the visual modulation of olfactory perception, there are less reports on the reverse. However, recently evidence was presented that olfactory input can indeed modulate visual perception. Fear-related chemical signals modulate visual emotion perception in an emotion-specific way (Zhou and Chen, 2009), while unpleasant odors reduce perceived attractiveness of faces (Demattè et al., 2007). Olfactory cues also bias the dynamic process of binocular rivalry: an odorant that is congruent with one of the competing images prolongs the time that image is visible and shortens its suppression time (Zhou et al., 2010, 2012). Finally, subliminal olfactory cues modulate visual sex discriminations made on the basis of biological motion cues: ambiguous point-light walkers are more often judged as males in the presence of unconsciously perceived male sweat (Hacker et al., 2013). Hence, there is now sufficient evidence for the modulation of visual perception by olfactory input.

### OLFACTION AND VISUAL ATTENTION

An organism continuously and simultaneously receives an overload of multisensory input from its environment. Because of limitations in processing capacity, simultaneous stimuli cannot be fully analyzed in parallel and thus compete for processing resources in order to gain access to higher cognitive stages and awareness. Attention serves as a gating mechanism to prioritize and enhance sensory information that is relevant for survival such as threats (Fox et al., 2002; Koster et al., 2004; Williams et al., 2006; Lin et al., 2009) or rewards (Anderson, 2013), while suppressing irrelevant information. Attentional selection is typically driven by stimulus saliency, novelty, and reward-related associations (Anderson, 2013). Attention acts upon and modulates information in each sensory modality (visual, auditory, olfactory, etc.; Woldorff et al., 1993; Zelano et al., 2005). Information from different sensory modalities is pre-attentively integrated into a unified coherent percept, resulting in multimodal internal representations in which attention can be directed (Driver and Spence, 1998). As a result, tactile (Van der Burg et al., 2009), auditory (Van der Burg et al., 2008), and olfactory (Seo et al., 2010; Tomono et al., 2011; Seigneuric et al., 2012; Chen et al., 2013; Durand et al., 2013) cues can boost the saliency of visual features, even when the cues provide no information about the location or nature of the visual feature. Thus, ambient odors (even at sub-threshold levels) can modulate visual attention (Morrin and Ratneshwar, 2000; Michael et al., 2003, 2005; Chen et al., 2013), even in 4-month-old infants (Durand et al., 2013). Recent studies have shown that odors can reflexively direct visual attention to *semantically congruent* visual objects (Seo et al., 2010; Tomono et al., 2011; Seigneuric et al., 2012; Chen et al., 2013). Objects that are semantically congruent with a presented odor are looked at faster and more frequently than other objects in a scene (Seo et al., 2010; Chen et al., 2013), even if participants are not aware that an odor has been presented (Seigneuric et al., 2010). It appears that crossmodal odor-object associations are automatically activated, without the need for explicit odor identification (Seigneuric et al., 2012), thus boosting the saliency of the corresponding visual object (Chen et al., 2013). Ambient odors also bias visual attention to favor stimuli that are *affectively congruent* to their hedonic quality (a case of affect-biased attention: Todd et al., 2012). Pleasant odors facilitate the processing of positive visual cues (Leppänen and Hietanen, 2003), while unpleasant odors facilitate the processing of negative cues (Ehrlichman and Halpern, 1988) and inhibit the processing of positive cues (Leppänen and Hietanen, 2003). The pre-attentive affective bias induced by ambient unpleasant odors probably serves the ecological purpose of facilitating threat detection (Krusemark and Li, 2012).

### CURRENT STUDY

The current study was performed to test if exposure to ambient odor can modulate the visual attention to signs of disorder in a desktop VE representing an urban area. Participants performed a walking tour through the VE while being exposed to either room air (control group), tar (typically perceived as unpleasant and frequently associated with burned or waste material), or the odor of freshly cut grass (typically perceived as pleasant and frequently associated with natural or fresh material). Whenever they noticed signs of disorder during their walk they reported their detection and their emotional response. The scent of cut grass had semantically congruent visual and auditory representations in the simulation, since the VE showed abundant greenery and contained the occasional sound of grass mowers in the associated soundtrack. The scent of tar could be associated with the occasional sounds of construction activities (e.g., hammering, sawing) in the soundtrack of the VE, and was affectively congruent with derelict areas in general. Since people tend to respond to an environment as a whole (a “molar” environment) rather than to its individual features (Bitner, 1992; Bell et al., 2010; Brosch et al., 2010; Houtkamp, 2012), and since affective qualities are prioritized in this categorization process (Brosch et al., 2010), the presence of an ambient scent with an affective (pleasant or unpleasant) loading was expected to bias the visual attention (away from or toward) for signs of disorder in the VE. More specifically, it was hypothesized that (H1) participants in the ambient tar (unpleasant) odor condition would report more signs of public disorder than participants in the control condition, because the unpleasant odor would bias visual attention to visual cues with a negative affective connotation. In contrast, it was expected that (H2) participants in the cut grass (pleasant) odor condition would report less signs of public disorder than participants in the control condition, because the smell of cut grass would bias their attention to the – semantically congruent – greenery and thereby distract them from the negative cues.

## MATERIALS AND METHODS

### VIRTUAL ENVIRONMENT

**FIGURE 1:**
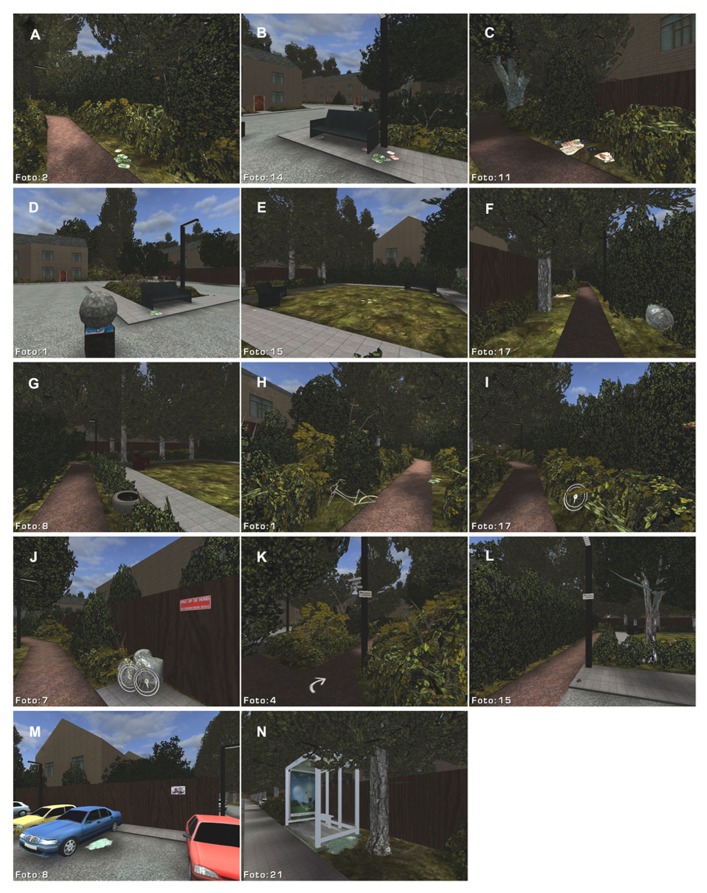
**Screen shots of the virtual environment, showing locations with litter (A–E), garbage (F,J), bicycle- and car parts (G–J), warning signs (J–M), cameras (K), and evidence of car burglary (M) and vandalism (N)**.

A small area in the town of Soesterberg, The Netherlands (with a rectangular shape and a total extent of about 200 m × 200 m; coordinates 52°; 7′ N, 5°; 17′34″ E:) was simulated in 3D using the Unreal Tournament 2004 game-engine v2.5 (Epic Games Inc.; for further details on the VE model and its contents see Toet and van Schaik, 2012). The area is enclosed by roads on four sides and contains blocks of houses, two squares with parking places, benches, and statues, two playgrounds with benches, and a network of pathways connecting the squares and playgrounds (see **Figure 1**). All houses have a garden in the back, typically enclosed with a wooden fence, with an exit door to a pathway. The pathways are typically covered with tarmac, and bordered on both sides with trees and shrubs. The houses are generally well maintained and quite uniform. The pathways and parks are reasonably well kept. The walking route (designated by arrows drawn on the ground) had no intersections and covered most of the area. To simulate a state of public disorder 42 test items were distributed over 34 different locations in the VE. The items signaled three different classes of social incivilities: Neglect (24 items), Vandalism (one item), and Crime (17 items: see **Table 1**; Perkins et al., 1992; Caughy et al., 2001), and had social connotations ranging from indifference (e.g., litter, trash, dog droppings) and loitering (e.g. empty beer cans, cigarette butts, fast food wrappers) to vandalism (broken bus shelter windows) and predatory crime (smashed car windows, crime watch signs, CCTV cameras, and camera surveillance signs).

**Table 1:** Experimental items, their connotations of physical and social disorder, and the experimental classification.

Experimental items (no.)	Social connotations	Experimental class (no. of items)
Garbage bags (2)	Neglect, indifference (Litter)	Neglect (24)
Cardboard boxes (1)		
Newspapers, flyers (2)		
Plastic shopping bags (2)		
Dog droppings (3)		
Bicycle frame (1)		
Bicycle wheels (2)		
Cigarette butts (1)		
Empty beer cans (7)		
Fast-food wrappers, boxes, paper cups (1)		
Old car tires (2)		
Bus shelter with broken windows (1)	Vandalism	Vandalism (1)
Smashed car windows and signs warning for car burglary (6)	Car burglary	Crime (17)
Neighborhood crime watch signs (3)	Home burglary	
Signs that homes are protected by private security services (2)		
Signs that homes are protected by dogs (2)		
CCTV security cameras and signs (4)	Predatory crime	


Numbers in brackets indicate the number of test items present in the VE.

The simulation was performed on Dell Precision 490 PC computers, equipped with Dell 19″ monitors. Logitech Rumblepad 2 Gamepads were used for navigation. User movement in the VE was from a first-person viewing perspective with walking motion supporting forward and backward movements and left and right rotation movements. User movement speed was fixed and collision detection enabled to prevent users from walking through objects. A non-repeating soundscape that was characteristic for the environment was composed from sounds (birds twittering, cars passing by, children shouting, hammering and drilling, and dogs barking) recorded at several locations and at different times in the corresponding real environment. The soundscape was presented through Sennheiser eH 150 headphones. A previous study showed that this soundscape effectively increased the ecological validity of the VE (Toet and van Schaik, 2012).

### ODOR SELECTION

The scent of freshly cut grass was selected as a semantically congruent pleasant odor in this study. This scent is generally considered to be stimulating and refreshing (the smell of freshly cut grass ranks among the top five preferred smell in several recent independent large scale polls in Britain: Reynolds, 2012; Henning, 2013). Since the VE used in this study shows a lot of grass and vegetation, the scent of grass may direct attention toward the greenery (Seo et al., 2010; Tomono et al., 2011; Seigneuric et al., 2012). The smell of cut-grass was created by mixing ethanol with cis-3-hexenol (leaf alcohol) in a 9:1 ratio. The associations that could be elicited by this scent in combination with the VE were investigated by presenting it to a panel of 10 participants while they were viewing the VE. The scent was presented in small glass tubes containing a cotton swab with three to four drops of the solution and sniffed by the participants approximately 5″ from their nose. About 9 out of 10 participants reported associations with greenery (four mentioned grass, three named freshly cut leaves and one mentioned broken twigs). All participants judged the scent to be pleasant.

An affectively congruent unpleasant scent was selected in a pilot test from a set of eight candidate aversive smells. The candidate smells were respectively Burned Wood (RS/420), Reptile (RS/424), Diesel Fumes (RS/423), Metal (RS/426), Dusty (RS/425), Tar (RS/401), Cow Manure, and Natural Gas (all obtained from RetroScent, Rotterdam, The Netherlands: ). The scents were identified by randomly assigned numbers, presented in small glass tubes containing a cotton swab with 3–4 drops of aroma oil, and sniffed by the 10 participants of the pilot test in random order, approximately 5″ from their nose, while viewing the VE. The degree to which each scent fitted the VE (how environmentally appropriate the scent was for the VE) was evaluated on a 11 point Likert scale (ranging from 0 = *absolutely not* to 10 = *definitely*). Tar received the highest mean score (7.4), followed by Dusty (5.7). In addition, although the exact the nature of the tar smell was not identified by any of the testers, 8 out of 10 spontaneously reported associations with fire and burned material, while it was unanimously judged to be a very unpleasant scent that could occur in an environment as the one represented by the VE.

A second pilot test served to investigate the spontaneous associations that may be elicited by the two selected scents (grass and tar) independent of visual feedback. Three small glass tubes containing a cotton swab with three to four drops of either the grass odor solution, the tar aroma oil or clear tap water were presented in random order to 10 participants (who did not take part in the first pilot test). The tap water condition served as a control condition. The participants sniffed the samples approximately five inches from their nose, and rated respectively their pleasantness and familiarity on five point Likert scales (ranging from 0 = *absolutely not* to 4 = *very much*). The grass smell received the highest mean pleasantness rating (3.6), followed by tap water (2.6), while the tar smell received the lowest mean pleasantness rating (0.2). The tar smell received the highest mean familiarity score (2.9), followed by tap water (2.0), and grass (1.9). For the tar smell, 6 out of 10 participants reported associations with smoke, fire, and burned material, while two participants associated this smell with industrial activities, and two others had respectively associations with garages and garbage dumps. For the grass smell, 5 out of 10 participants reported associations with nature, flowers, pine trees, or leafs, one was reminded of fruit, while four participants associated it with air refreshers or cleaning material. Hence, the tar smell was frequently perceived as an unpleasant smell and associated with negative (burned or waste) material, while the grass smell was predominantly considered a pleasant smell associated with positive (natural) material.

### ODOR DIFFUSION

Scents were diffused in the room (about 25 m^2^) through a commercial electronic dispenser (1-3 RS-Classic Scentvertiser, RetroScent, Rotterdam, The Netherlands: ). No odor was applied in the control condition. The dispenser was placed out of the participant’s sight behind a screen. The participants could not hear the sound of the dispenser when they wore their headphones and listened to the soundscape of the VE. The experimenter turned on the dispenser after the participants had started their tour through the VE and he turned it off before they were instructed to take off their headphones. Odor was intermittently diffused (with a cycle period of 1 min) during the experiment so that the participants received fluctuating concentrations over time, thus preventing full adaptation.

It is likely that both aversive and pleasant odors turn on the sensory-driven attentional systems even at subthreshold levels to facilitate the detection and analysis of behavioral relevant stimuli (Krusemark and Li, 2012). In this study olfactory stimulation was therefore intentionally performed at a near-threshold level to preclude the possibility of top-down influence on visual perception (e.g., the use of explicit search strategies), thereby narrowing the effects down to bottom-up sensory driven attentional systems facilitating threat or reward detection. Ideally, the odor intensity should be sufficiently strong to be just noticeable when attended to. The odor intensity used in this study was between low and intermediate, corresponding to a mean level between 3 and 5 on a 10-point scale. A pilot experiment was performed to determine a setting of the dispenser and a duty cycle that resulted in a mean rating of 5.

The room in which the test was performed was well ventilated prior to each session. Only one scent per day was diffused to avoid mixing odors, and the lab was fully ventilated overnight to remove any lingering trace of the scent. Before beginning the study each morning, the room was “sniff-tested” by the two experimenters; no odors were detected to have remained in the room.

### INSTRUMENTS

#### General questionnaire

As the results may be influenced by the characteristics of the participants, they were asked to complete a *General Questionnaire* including socio-demographic measures (sex, age, and education). Education was clustered into four groups: middle and higher level education, academic education, and other types of education.

#### Mental state questionnaire

A 7-item *Mental State Questionnaire* (adapted from Spielberger, 1983), consisting of four negative (*agitated, angry, anxious, distressed*), two neutral (*calm, relaxed*), and one positive (*cheerful*) emotional terms served to assess the emotions elicited by the individual incivilities. On each encounter with a sign of disorder during their walk participants reported their emotional reaction by selecting one of the seven items (“*I feel⋯*”).

#### Post-experiment questionnaire

A 4-item *Post-Experiment Questionnaire* contained three questions investigating the extent to which the ambient temperature, illumination, and atmosphere in the room were characteristic for the VE (these three items were scored on a 5-point Likert scale, ranging from 1 = *completely disagree* to 5 = *completely agree*) and an open question (“*Was there anything else you noticed during the experiment*?”) to test if the participants had noticed the ambient scent in the room.

### EXPERIMENTAL PROCEDURE

After their arrival at the laboratory, the participants first read and signed an informed consent form. Next, they filled out the *General Questionnaire*. Then they read the following instructions:

“*The experiment concerns an area of Soesterberg near the TNO lab, and will take about 45 minutes. Citizens living in this area are concerned about the increasing social disorder in their neighborhood. They intend to draft a plan of action to confront this problem. After making an inventory of the different types of incivilities occurring in their neighborhood, the citizens will prioritize the order in which these should be addressed. To enable a large number of people to give their opinion on the social disorder in this area, the concerned citizens have commissioned a realistic and highly detailed computer model of their neighborhood.*

It is your task to make a tour through this virtual model and assess the social disorder in this neighborhood. Your route is marked by arrows drawn on the ground. Each time you notice signs of incivilities (e.g., litter, dog droppings, broken car windows, etc.) during your inspection tour, you are requested to:

1. *Make a snapshot of each sign of incivilities you notice (by pressing key F12).*
2. *Enter a brief description of the incivility on your questionnaire.*
3. *Report your current mental state by choosing one of the 7 emotional terms on the ‘Mental State Questionnaire’ (agitated, angry, anxious, distressed, calm, relaxed, cheerful).”*

Next, the experimenter verified if the participants had understood their instructions, and started the simulation. The experimenter then explained the function of the gamepad, and gave the participant the opportunity to practice maneuvering through the VE for about 5 min. At the end of this practice period the experimenter checked if the participant was able to perform the required maneuvers, and whether the participant paid attention to the arrows on the ground and the signs of disorder. Then, the experimenter gave the participants the printed questionnaires which they could use to fill out their reports, and positioned the point-of-view in the VE at the starting location, facing the direction of the route. The participants then put on their headphones and started their walkthrough, which they performed at their own pace. Each time the participants noticed signs of disorder during their walk they reported the item they had noticed and their current mental state. During the test, the experimenter was seated behind a screen in the room and intermittently turned on the odor dispenser at one minute intervals, maintaining a slightly fluctuating near threshold ambient odor level. Finally, after finishing their walkthrough, the participants filled out the *Post-Experiment Questionnaire*.

The experimental protocol was reviewed and approved by the TNO internal review board on experiments with human participants (TNO Toetsings Commissie Proefpersoon Experimenten, Soesterberg, The Netherlands), and was in accordance with the Helsinki Declaration of 1975, as revised in 2000 (World Medical Association, 2000). The participants provided their written informed consent prior to testing. The participants received a modest financial compensation for their participation.

### PARTICIPANTS

The experiment was performed by 69 participants (3 groups of 23 each) that were selected from the TNO database of volunteers: 39 males and 30 females, aged 43 ± 18 years. The selection criteria guaranteed that they were not familiar with the urban area represented by the VE, that they had no problems with their sense of smell, and that they all had normal (or corrected to normal) vision with no color deficiencies. Also, they were unaware of the aim of the experiment. The participants’ mean age, level of education, and computer proficiency and game experience were approximately the same for all three (no-ambient smell, ambient tar odor, and ambient grass odor) experimental conditions.

### DATA ANALYSIS

The emotional responses reported for the detected signs of disorder (from the *Mental State Questionnaires*) were clustered for each of the three classes of experimental items: neglect, vandalism, and crime. Analysis of variance (ANOVA) was used to test the relationships between the main variables. Chi-squared tests were performed to determine whether observed frequencies were significantly different from expected frequencies. The statistical analyses were performed with IBM SPSS 20.0 for Windows. For all analyses a probability level of *p* < 0.05 was considered to be statistically significant.

## RESULTS

Chi-squared tests showed a significant difference (χ^2^ = 18.94; df = 4; *p* ≤ 0.05) between the observed and expected frequencies of the emotional responses (negative, neutral, or positive) associated with the reported items (signs of incivilities) in the classes Neglect, Vandalism, and Crime. Items in the classes Vandalism and Crime were more frequently associated with negative emotional responses than items in the class Neglect.

**FIGURE 2:**
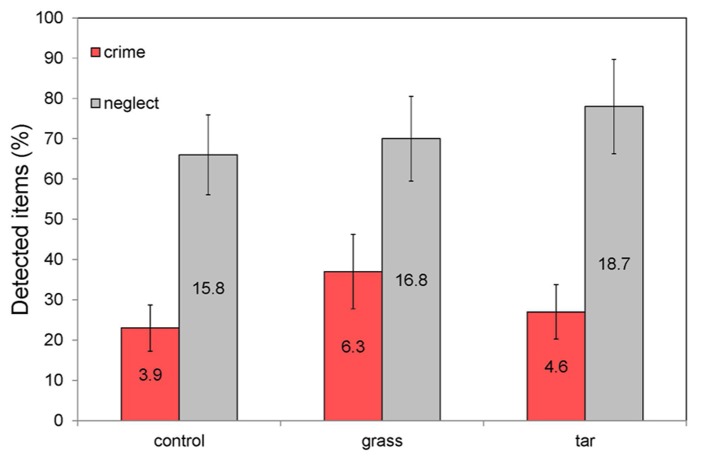
**Percentage of detected items signaling* Neglect *(a total of 24 items)**and* Crime* (a total of 17 items) in each of the three experimental ambient scent conditions (no odor, grass odor, tar odor).** The labels inside the bars represent the mean number of detected items. The error bars represent the standard error in the mean.

**Figure 2** lists the detection performance for items signaling* Neglect *and* Crime* in each of the three experimental conditions. To enable a comparison of the performance between the different experimental classes (that were each represented by a different number of test items) the results are expressed in percentages (for the sake of completeness this figure also provides the mean number of detected items for each condition). **Figure 2** clearly shows that the relative detection performance is lower for signals of crime than for signals of neglect in all conditions.

A one-way ANOVA showed that the mean numbers of detected items signaling**respectively* Neglect *and* Crime *did not differ significantly**between the three ambient odor conditions. More specifically, there were no significant differences between the *Control* and *Grass* (respectively *F*_1,42_ = 0.57, *p* = 0.45 and *F*_1,37_= 1.76, *p* = 0.19), *Control* and *Tar* (respectively *F*_1,45_= 3.10, *p* = 0.09 and *F*_1,36_= 0.96, *p* = 0.33) and between the *Tar* and *Grass* (respectively *F*_1,42_= 0.79, *p* = 0.38 and *F*_1,38_= 0.01, *p* = 0.93) conditions. Hence, the hypotheses (H1 and H2) that participants in the (un)pleasant odor condition would notice (more) less signs of disorder than participants in the (odorless) control condition is not supported by the present data. Compared to the control (odorless) condition, participants reported the same mean number (percentage) of signs of disorder in both (tar and grass) ambient odor conditions. In addition, there appears to be no effect of the hedonic tone of the ambient odor on visual attention toward neglect or crime objects. Also, ambient scent did not affect participants’ subjectively reported emotional state. Since there were no main or interaction effects of age and level of education, these factors were omitted from later analyses.

The VE contained multiple objects representing* Neglect *and* Crime, *but**only a single object signaling *Vandalism* (a broken bus shelter). Since this item was rather conspicuous it was never missed by any of the participants. Hence, the results for this item have no discriminative value and are therefore not further discussed in this study.

In response to the open question in the *Post-Experiment Questionnaire* one participant (out of 23) claimed to have noticed a Lysol smell in the room in the control condition. In the tar odor condition one participant (out of 23) reported to have noticed a smell, but he was unable to identify its nature, and did not link the odor to the exploration task. No participant noticed a smell in the grass odor condition.

## DISCUSSION

Based on the present we cannot conclude whether a subliminal ambient scent can affect the perception of the VE. The finding that ambient scent did not seem to affect participants’ subjectively reported emotional state agrees with similar findings from related earlier studies who observed that pleasant ambient scents did not affect self-reported mood and arousal (Morrin and Ratneshwar, 2000, 2003; Teller and Dennis, 2011).

Contrary to our expectations the presence of the ambient odors also did not bias the participants’ attention for the experimental items. Thus, we found no indication that ambient smell of a given nature selectively biases visual attention to details in a desktop VE. The design of the current study does not allow to determine whether the fact that we did not observe an effect is due to (1) the absence of an effect or (2) the limited power of the study design itself. In any case, it appears that ambient smell may only have limited effectiveness as a tool to direct a user’s attention to specific details in a desktop VE. This result is somewhat surprising given the substantial amount of evidence that odors draw visual attention to congruent visual objects (e.g., Seo et al., 2010; Tomono et al., 2011; Seigneuric et al., 2012; Chen et al., 2013). However, the present result agrees with earlier reports that ambient scent has no effect on shopping behavior (Schifferstein and Blok, 2002; Teller and Dennis, 2011). It has in fact been argued that previous reports of significant effects of ambient scents on perception, emotions, and behavior in shopping environments need to be taken with care since most previous studies typically did not control for different sources of bias (Teller and Dennis, 2011). Our results also agree with those of Schifferstein and Blok (2002), who found that the scent of freshly cut grass did not affect sales of thematically (in-) congruent products. They argue that ambient scent is probably more diagnostic for the physical environment of the observer than for the particular items in that environment. This suggests that ambient scent may only effectively guide visual attention when there is a close link between the affective or semantic qualities of the scent and visual features in the VE. Although there may be a semantic link between the scent of cut grass and the greenery shown in the VE, the link between the scent of tar and signs of disorder is probably less evident. Also, more immersive VEs may be required to automatically establish associations between ambient scents and the VE itself. In case of desktop VEs, a close spatiotemporal link between the contents of the desktop VE and the scents with which they are supposed to be associated may be required to effectively establish diagnostic associations (i.e., smells and visual features may need to appear and disappear together to effectively induce the illusion that the smells actually emanate from the objects shown on the screen) that guide a user’s attention.

Experimental items signaling vandalism (e.g., a damaged bus shelter) and crime (e.g., home protection signs and cameras) more frequently evoked negative affective appraisals than items representing neglect (e.g., litter, dog droppings, old bicycle parts). This finding agrees with the discriminant validity of different types of perceptual incivilities that is also found in the real world (e.g., between crime and social incivilities: Worrall, 2006; Armstrong and Katz, 2010). In reality, signs of crime are also more likely to evoke negative appraisals since they are typically associated with the risk of personal victimization (Phillips and Smith, 2004). This finding suggests that the affective appraisal of the VE had at least some ecological validity.

In all experimental conditions, the relative detection performance for signals of crime was lower than for signals of neglect. This is probably due to the fact that most signals of crime (i.e., the warning signs and CCTV cameras) were positioned at eye height or higher in the VE (e.g., attached to trees, lamp posts, or walls), while the signals of neglect were on the ground or on low supports (statues). Although participants were informed about the nature of the signals of disorder, and shown examples during their introduction to the experiment, they may have focused primarily on the signs of neglect on the ground and may have paid less attention to signals higher up in the scene. The fact that the walking route was indicated by arrows drawn on the ground may also have induced a bias for downward perception.

Summarizing, the present study does not allow us to conclude whether ambient odors may be an effective tool to direct a user’s attention to specific (congruent) objects in a desktop VE (e.g., by evoking implicit associations).

### LIMITATIONS OF THE PRESENT STUDY

In previous studies on the effects of odor on visual attention participants freely inspected visual scenes without any explicit instructions, and odor induced attentional bias became manifest in spontaneous fixation behavior (Seigneuric et al., 2010; Seo et al., 2010). In the current study the participants were explicitly instructed to look for signs of disorder in the VE. The cognitive effort associated with this strict assignment may have overruled any odor induced attentional bias effects. However, the fact that only a fraction of the targets was actually noticed suggests that there was still room for odor modulated performance enhancement.

The walking route through the VE was indicated by arrows drawn on the ground, which may have induced a bias for visual search near the ground. Unfortunately, fixation behavior was not measured in this study, so this hypothesis cannot be verified.

The scent of grass had an explicit visual representation in the VE, while the scent of tar could only implicitly be linked to visual (litter) and auditory (construction sounds) elements in the VE. Future studies should preferably employ scents that have explicit and unequivocal visual counterparts in the VE. Also, a range of both (1) neutral odors or odors with the same valence but different semantic connotations, and (2) odors of different valence but without any semantic counterparts in the VE should be deployed to enable a distinction between effects induced by hedonic or semantic congruency.

There was only one sign of vandalism in this study (the broken bus shelter) which was also highly salient. As a result this item had no discriminant value. Future studies should include a larger number of test items for each experimental class, with different (including low) visual saliencies. The attention enhancing effects of olfactory cues may be more prominent for targets with low visual saliencies.

The participants in this study reported that they had no problems with their sense of smell at the time of this experiment. Also, there were no entries in the TNO database of volunteers that any olfactory deficiencies had been noted during their participation in previous smell experiments. However, since we did not explicitly test their sense of smell in the current experiment there is no guarantee that they all had normal olfactory function.

### SUGGESTIONS FOR FUTURE RESEARCH

It would be interesting to test whether the finding that specific odors can reflexively direct visual attention to *semantically congruent* visual objects (Seo et al., 2010; Tomono et al., 2011; Seigneuric et al., 2012; Chen et al., 2013) can also be replicated with dynamic desktop VEs. To effectively guide a user’s attention dynamic olfactory displays are probably required so that a close spatiotemporal link may be established between the contents of the VE and the scents with which they are supposed to be associated.

Future studies should also register eye movements, since human fixation behavior may provide valuable additional information to subjectively reported results. Also, future studies should track the exact path of the participants through the VE. It is in principle possible that participants use scent cues to adjust their distance to certain items in the VE (e.g., that they show an approach or avoidance behavior, maintaining a larger distance to unpleasant smelling items, and coming closer to pleasant smelling items). Since distance affects the visual saliency and detectability of targets this may affect the results. Path deviations are not likely to be a significant confounding factor in the present study, since most parts of the route were rather narrow and did not leave much room for deviations.

It has previously been shown that the addition of olfactory cues to an immersive VE increases the user’s sense of presence and perceived realism of the simulated environment, and ultimately his memory for details therein (Dinh et al., 1999; Washburn et al., 2003; Tortell et al., 2007). It would therefore be interesting to investigate whether an odor induced visual attention bias may also become manifest for desktop VEs when memory for details is tested instead of the number of detections. From the abovementioned previous studies we expect that participants in an (un)pleasant odor condition will remember (more) less signs of disorder than participants in an odorless control condition after completing their inspection tour of the VE.

## Conflict of Interest Statement

The authors declare that the research was conducted in the absence of any commercial or financial relationships that could be construed as a potential conflict of interest.
